# Collectanea Medica: Consisting of Anecdotes, Facts, Extracts, Illustrations, &c.

**Published:** 1820-08

**Authors:** 


### COLLECTANEA MEDICA:

CONSISTING OF

ANECDOTES, FACTS, EXTRACTS, ILLUSTRATIONS, &c.

*Relating to the History or the Art of Medicine, and the Auxiliary Sciences.*

Floriferis ut apes in saltibus omnia libant,
Omnia nos itidem depascimur aurea dicta.

### *Observations on the Consequences of the Section of the Two Nerves of the Eighth Pair in the Horse.* By M. DUPUY, Professor at the Royal Veterinary School at Alfort.

[From a Memoir read to the Medical Society of Paris, March 21, 1820.]

TWO different modes of proceeding have been followed in making these experiments on the division of the par vagum: the first consists in making the section of the nerves in the middle of the chest; the animal then dies a few hours afterwards, the same way as in asphyxia from privation of air. Horses thus operated on make a noise in breathing, like those which are called *broken-winded.* In the second, an opening is made in the trachea in the middle of the first and second cartilaginous circles. Immediately after, an incision is made on each side of the neck, in the direction of the eighth pair of nerves, which are isolated, and a ligature is passed under them, but left loose. The horse is then released from the shackles in which he had been placed, in order that the operation might be performed without danger and difficulty, and he is then placed in the stable. On the following day the nerves are drawn without the wounds in the neck by means of the ligatures, and the division of them effected. By this proceeding, one horse died on the fifth, the others on the ninth day, presenting phenomena which I shall rapidly describe, in relating what we observed in an English horse, six years old and very strong, though affected with the glanders.

During the first day, we remarked that the skin of the neck and head was moistened with sweat; that the temperature of those parts was increased, on comparing it with that of the posterior parts of the body; the pupil was dilated; convulsive movements were manifest in the subcutaneous muscles, accompanied with general rigors; and, after the animal had eaten a part of his food, he was affected with the gripes very severely. He pawed the ground with his fore-feet, changed his place every instant, and laid down only to rise up again instantly. The breathing was laborious and sonorous, and the pulse full and accelerated. He continued to eat at different times during the day; and towards the evening we observed both the liquid and solid aliments which he took pass out through the opening in the trachea, as he swallowed them.

On the second day, the same things were observed as on the preceding evening. The edges of the wound made in the trachea were tumefied, painful, and emphysematous.

On the third day, signs of peripneumony became evident. Respiration was short and accelerated; the pulse frequent, rapid, and hard. The animal was then bled, which relieved him, and he began again to eat; but soon became affected with the gripes.

On the fourth day, the animal had general rigors, after having drank four gallons of water at the ordinary temperature. No portion of this water passed out through the wound in the trachea.

On the fifth day, nothing particular was observed: however, the pupil continued always dilated, and the head covered with sweat.

On the sixth day, he was troubled with a continual cough, which much fatigued him. He was bled in the jugular vein, with the intention of diminishing the irritation. Although he had taken less food than in the preceding days, a large quantity of it escaped from the opening in the trachea, and he was also tormented with the gripes.

On the seventh and eighth days, there were but slight changes in the state of the animal: he however appeared to be much weaker. The ends of the divided nerves were tumefied, red, and exhaled a fetid odour.

On the ninth and last day, respiration was difficult and embarrassed; the trachea and the bronchiæ contained a considerable quantity of fetid puriform matter. The animal fell in the afternoon, as it were, suffocated: on making attempts to rise again, a large quantity of puriform fluid escaped from the trachea, and he died towards the evening, after having suffered considerably.

On dissecting the body, we found the œsophagus filled and distended by greenish fibrous matters, which were dry and heaped together in that canal. In the stomach, matters of the same nature, and in a large quantity, were also accumulated in hard masses: they exhaled a very fetid odour.*

The small intestine was empty; the coecum and colon only contained about sixteen pounds of dry yellowish matter, instead of from sixty to eighty pounds, which are ordinarily found in them.

The tissue of the lungs was of a violet colour: those organs were voluminous, engorged with black blood; hepatized to a great extent, as is remarked after severe peripneumony. The bronchiæ contained a brownish sanious matter, of a very fetid odour. This matter came from several vomicæ which opened into those canals.

The brain and spinal marrow were of a less firm consistence than ordinary. The ends of the divided nerves were red, tumefied, and the

---

* Prof. Dupuy has not stated what the horse took as food after the division of the nerves, nor what he considered the matters to be which filled the stomach and œsophagus. We must suppose them to have been hay, altered by being collected in the stomach without undergoing the digestive process, and dry, because the secretion of the gastric fluid had ceased, in consequence of the division of the nerves; circumstances according with the results of the experiments of Dr. Wilson Philip.—Edit.

pulp of them, impregnated with blood, exhaled an odour nearly similar to that of carious bone.

We have repeated these experiments on several horses, and have obtained analogous results.

As we have already stated, when the first proceeding is followed, the animal dies asphyxiated in a few hours. We may account for this result, by considering that the inferior laryngeal nerves which are distributed to the muscles which dilate the larynx are divisions of the eighth pair, and consequently those muscles are paralyzed by the division of these nerves in the middle of the neck; whilst the superior laryngeal nerves, which have another origin, are distributed to the constrictor muscles, which preserve all their activity, and, closing the sides of the glottis, prevent the passage of the air into the lungs. On making an opening, then, into the trachea, we may conceive that death should not occur equally soon: however, as the circulation through the lungs is rendered languid, engorgement of those organs takes place, the change of the blood from arterial to venous is suspended, and then at length the animal perishes.

In the course of some experiments made a few years since, two ounces of nux-vomica, rasped and made into a ball, were introduced into the stomach of a horse, in whom the eighth pair of nerves had been divided, without producing any particular effect; whilst the same quantity, given to another horse which had not undergone that operation, destroyed it in a few hours, after three terrible fits of violent convulsions and tetanic spasm.

After having practised merely tracheotomy on another horse, we injected, by this opening, half a pint of brandy of ten degrees strength into the bronchiæ: all the signs of drunkenness appeared; but ceased at the end of seven or eight hours.

These experiments will serve to account for what occurs in horses that are called broken-winded. We have indeed seen one of these horses, in which a cancerous tumor was found at the entry of the thoracic cavity, pressing strongly on the eighth pair of nerves, above the origin of the recurrent nerves. The trachea was in the ordinary state.

Another horse, affected in the same way, had a large, hard, and schirrous engorgement of the lymphatic glands, situate at the division of the trachea into the bronchiæ, and compressing the recurrent nerves. We may then, we believe, conclude that this affection may be attributed to compression or to paralysis of the recurrent nerves, and which are distributed, as M. Magendie has demonstrated, to the dilator muscles of the larynx. On cutting one of the eighth pair of nerves in a horse entirely through, and the other incompletely, we have caused the animal to make a noise in breathing similar to that made by horses called broken-winded.

### *On Predisposition to Disease.**

[From Mr. Pring's *Indications which relate to the Laws of the Organic Life.*]

The growth of the body, and its changes, are similar to those of the mind; and the former may be further illustrated by what is more familiarly observed of the latter. The mind of an infant has been said to be a blank tablet: this is said in the way of metaphor. The mind in this early stage is a state of properties which has certain relations with the external world; the effect of these relations is, that the mind, which was a *predisposition*, is made, by a common act of causation, another identity, by the introduction or combination of additional causes; or becomes, according to common metaphorical language, stored with ideas, which again are related with each other, and help to accomplish the phenomena of association and inference, &c. Thus much for our present purpose: we see this mind by degrees making its acquisition of knowledge; as its identity changes, new relations are opened; its notices are first on one set of objects; having attained the state which these constitute, its notices are extended to another set of objects; these familiarized, its *predisposition* is again a new one, and it takes cognizance of things to which it was before blind or unconscious. By this progression, similar to that which all nature observes, one acquisition disposes to another, materials are accumulated, new associations arise, thought improves, taste is developed, and the interchange of agencies in the constituents of mind itself or its affections, by relation with the external world, are limited or extensive; confined to the sober walk of industry, plodding upon business or upon domestic concerns; or, if it is so prepared, taking a wider range, or soaring perpetually some bold flight of genius. These processes make a growing predisposition, which can never preserve one given state, because, the constituents of mind being related, and these relations being further extended to all the variety of externals, progressive changes must succeed; and these are rapid or slow, not necessarily in their own nature, for in this they are perhaps uninterrupted, but in the apparent and in the imperceptible conversions of related beings. If then it be asked why the testes secrete at sixteen, and did not secrete before; let it be asked as a parellel, why a person understands a certain proposition in Euclid at sixteen, and did not before? The reason is the same: a progressive change or a series of causation leads up to this effect. The character of the changes of mind, each particular phenomenon which it exhibits, is that which it is made by its own constitution or by an external related agent; each change, each phenomenon, whether belonging to the physiology or disease of the body, is produced from similar sources. We have then to discriminate be-

* The rapid manner in which we were obliged to pass over the work of Mr. Pring, in our review of it in the last number of this Journal, prevented our giving any thing more than an imperfect hint of the author's manner of treating this interesting subject: we therefore transcribe this passage on the present occasion, in order to remedy, in some degree, the imperfections of our former sketch of the character of the work from which it is selected.—Edit.

tween those things which proceed from the original constitution, or parental radicle, and those which are to be attributed to the influence of externals.

It is demonstrated by our anatomy, that the first perceptible original of an animal (as of a man) is an insignificant aggregation of matter, possessing no sensible arrangement, but endowed with certain properties. This radicle suffers or exhibits the numerous changes which are just represented; but these changes do not occur among its own inherent properties, but in obedience to a relation which subsists between these and certain external properties and substances. The question here, then, to be discussed is, what share have the properties of the ovum in the subsequent changes which it undergoes?

There are two modes in which the properties of the ovum may contribute towards the conversions which occur in its development. 1. The first state of the properties of the ovum may be so related with the external as to produce, in conjunction, one change; this change, or this new state, may open a new relation with externals, and produce another change; and so on through a series: in this way the properties of the original predisposition, or the ovum, may have, with respect to subsequent phenomena, the importance only of remote causes; and the series and nature of ensuing changes, though taking their determinate character from this first predisposition, may concern principally the accession of properties and substances obtained from the external world. The second mode in which the original properties of the ovum may operate in regard to ensuing changes, is by a direct causation with respective phenomena; as if that progressive change which has been described went on in properties originally belonging to the ovum, and as if every new change resulted from the development of a latent property, in the manner explained. The latter of these has been preferred, in the instances there cited, in our section on the constitution of the ovum. As we possess no criterion by which we may discriminate when, in particular instances, either of these modes obtains; as also it is probable that in all instances these original and external properties are mixed in causation, and the more especially as, in our present state of knowledge, we may rest satisfied with a deduction from alternatives; on these accounts it appears superfluous to state the objects with which an inquiry for such a criterion might be conducted. Let us therefore, as such is the highest success to which we might aspire at this time, be contented to state our deduction, or corollary, respecting the share which the first properties of the ovum have in subsequent phenomena, whether of health or of disease.

If the original properties of the ovum have, with respect to the subsequent processes, the importance only of remote causes, then the nature, order, &c. of such changes are determined by these properties of the ovum, although the respective effects which occur in catenated causation may be constituted by properties acquired from the external world. And, whatever differences might occur in the development of two animals of the same species, they are to be referred to an original peculiarity in the respective ova, which, being differently constituted, run through a series of different relations, and of course exhibit different phenomena. If the processes of development, &c. are to be attributed to progressive change going on between properties originally possessed by the ovum, each property, as it passes from the latent to the active form, producing as a constituent its own effect, then also the phenomena of development, conversion, &c. are to be attributed to the original constitution of the ovum: in the first case, this constitution is preparatory to subsequent occurrences; in the second case, they are contributary, or have the importance of a real cause, which is that without which the effect cannot exist. We have, it is said, no satisfactory criterion by which either of these alternatives may, in particular cases, be positively adopted.

We observe in the intellectual system, that its phenomena concern chiefly the acquired properties, viz. the ideas which are acquired, or formed, with the help of the intellectual predisposition. We see also that the information of the mind, and its peculiarities in individuals, take their determinate character from the intellectual predisposition: it is this which directs and produces all the variety of opinion and the infinite motives to action in different individuals, surrounded by the same externals, or existing in nearly similar circumstances. Yet here there is similitude in the offspring to an original in the parent, as in the corporeal phenomena. The testes begin to secrete at sixteen; a person becomes consumptive at five-and-twenty, whose mother died of consumption perhaps at about the same age; a person at three-and-thirty becomes deranged in mind, whose father or grandfather was deranged at about the same period. Here it would appear as if properties, in all the cases which could specifically determine these events, belonged to the ovum, and remained latent until they were developed by that progressive causation which we have attempted to describe. On the other hand, we know that insanity engages principally the acquired properties of the mind or its ideas; for the form of insanity may consist in disordered judgment or inference, and perhaps only on one subject, the act of which consists in the comparison of ideas, from the analogy of which an inference is made: or the form of insanity may be one of disordered association only; comparison, or association, of what?—of ideas, or of that which is not obtained from parents, or possessed by the ovum. It is perfectly clear that, if a man were born of mad parents, and had in ever so strong a degree the hereditary taint, he never could have either of the above forms of insanity, viz. of disordered comparison, or of disordered association of ideas, if he were so circumstanced from his birth as to be precluded the acquisitions from externals, as, if he were enclosed in a dark room and never permitted to hear an articulate sound. Yet in these forms of insanity the predisposition is hereditary, although the form itself is principally constituted by the acquirements from without, and dependant perhaps upon education and a long train of connected causation. In this case, although the acquired properties or ideas have a principal share in immediately constituting the insanity, and in determining the consequent acts of volition, yet we must suppose the predisposition to ideas to have a share, or, in fact, if it is a principle of consciousness, to be the basis of their individual existence. Here then we may look for the cause, or a cause, of the insanity; and, as the insanity cannot arise out of the mere predisposition, but is principally dependent upon relations of ideas, so in this, as in the corporeal instances, we perceive the probability that the original and the acquired properties are mixed in the causation of progressive phenomena; and in this case too, as in the other, we perceive the impossibility of pronouncing; such a share have the original, and such the acquired, properties in these occurrences. We must for the present be content with our alternatives.

From this view, it appears that we cannot fix a period to the commencement of the processes which are preparatory to disease: the necessity of a progressive change leading up to this state of disease, is demonstrable; and, with respect to the causes which constitute the disease, we cannot lay down any general criterion by which to distinguish whether they belonged to the ovum, or whether introduced from without. In cases of contagious and infectious disease, &c. we know that a cause is obtained externally; but the share of this cause and that of the original properties, as we may conjecture that they produce effects in conjunction, we cannot even here discriminate.

That state of the properties which is the stage of progressive change immediately preceding disease, is called predisposition; any change, the tendency of which is finally to impair functions, &c. may be said to comprise a diseased state. But there is convenience in the distinction between predisposition and disease: according to our notion of the difference between these two, predisposition is a change of the state of perfect health which does not produce symptoms; disease is also a change of the state of health, but it is manifested by symptoms: the former change is not related with our faculties of perception, &c. the latter change is related with our faculties of perception. The most healthy state may be predisposed to disease, because there is no state which does not somewhere find a relation capable of producing change with some or other external. Thus all bodies are predisposed to disease and death by the relation of their properties with arsenic; nearly the same thing may be said of the plague, of morbid poisons, &c. But it is proper to confine the use of the term *predisposition* to those states which have a relation productive of disease with natural and habitual properties, or with those to which the same subject, at other times, and the rest of the species, may be regularly or occasionally exposed, without the supervention of disease.

